# Novel tri‐isotope ellipsoid approach reveals dietary variation in sympatric predators

**DOI:** 10.1002/ece3.5779

**Published:** 2019-11-04

**Authors:** Christina Skinner, Aileen C. Mill, Steven P. Newman, Jason Newton, Matthew R. D. Cobain, Nicholas V. C. Polunin

**Affiliations:** ^1^ School of Natural and Environmental Sciences Newcastle University Newcastle UK; ^2^ Banyan Tree Marine Lab Vabbinfaru Republic of the Maldives; ^3^ NERC Life Sciences Mass Spectrometry Facility Scottish Universities Environmental Research Centre East Kilbride UK

**Keywords:** coral reef, foraging, individual specialization, stable isotopes

## Abstract

Sympatric species may partition resources to reduce competition and facilitate co‐existence. While spatial variation and specialization in feeding strategies may be prevalent among large marine predators, studies have focussed on sharks, birds, and marine mammals. We consider for the first time the isotopic niche partitioning of co‐occurring, teleost reef predators spanning multiple families. Using a novel tri‐isotope ellipsoid approach, we investigate the feeding strategies of seven of these species across an atoll seascape in the Maldives. We demonstrate substantial spatial variation in resource use of all predator populations. Furthermore, within each area, there was evidence of intraspecific variation in feeding behaviors that could not wholly be attributed to individual body size. Assessing species at the population level will mask these intraspecific differences in resource use. Knowledge of resource use is important for predicting how species will respond to environmental change and spatial variation should be considered when investigating trophic diversity.

## Graphical abstract

Food web dynamics are driven by resource availability, and sympatric species may partition resources to facilitate co‐existence. Using a novel tri‐isotope ellipsoid approach, we consider for the first time the isotopic niche partitioning of co‐occurring reef predators spanning multiple families. We demonstrate substantial spatial and intraspecific variation in resource use that is not attributable to body size. Assessing species at the population level may mask intraspecific differences in resource use where predators play vastly different ecological roles. Furthermore, knowledge of resource use is important for predicting how species will respond to environmental change.

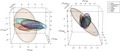

## 1 INTRODUCTION

Trophic interactions are key regulators of community dynamics and ecosystem function. Food web and population dynamics are driven by resource availability, with sympatric species often in direct competition with each other (Schoener, 1983). Resource partitioning often occurs among co‐occurring species to reduce inter‐ and intraspecific competition when resources are limited (Schoener, 1974). Often linked to body size or ontogeny (Werner & Gilliam, 1984), increasing evidence suggests that individuals may vary in their resource usage compared with conspecifics of the same age and size (Araújo, Bolnick, & Layman, 2011). As trophic energy dissipates up food webs, food resource scarcity is likely to be an important driver of foraging behavior in large predators. Consumers may alter their foraging to include underutilized resources when competition is high, leading to dietary specializations within populations (Bolnick et al., 2003).

Predators (here referring to upper trophic level sharks and teleosts) are thought to play an important role in structuring communities. Through their foraging, they may alter prey behavior (Lima & Dill, 1990) and, being more mobile, may couple distinct food chains (McCauley et al., 2012), altering energy flows and stabilizing food webs (McCann, Rasmussen, & Umbanhowar, 2005; Rooney, McCann, Gellner, & Moore, 2006). Feeding specializations have been extensively documented in upper trophic level vertebrate populations, particularly fishes (Araújo et al., 2011; Bolnick et al., 2003). While marine predators are often considered to be dietary generalists (Costa, 1993; Gallagher, Shiffman, Byrnes, Hammerschlag‐Peyer, & Hammerschlag, 2017), they may vary significantly in their trophic ecology at both the individual and species levels. Such specializations can alter community dynamics (Bolnick et al., 2011), so species‐level assessments of trophodynamics will not account for differing ecological roles (Matich, Heithaus, & Layman, 2011).

Stable isotope ratios in animal tissues provide unique dietary perspectives and reveal important facets of resource use (Bearhop, Adams, Waldron, Fuller, & Macleod, 2004) as they reflect assimilation of prey material into consumer bodies over time (Post, 2002). Carbon (δ^13^C) and sulfur (δ^34^S) isotope data help elucidate the production sources responsible for the energy flow in the food web, while nitrogen (δ^15^N) suggests the relative trophic position at which an animal is feeding (Connolly, Guest, Melville, & Oakes, 2004; Croisetière, Hare, Tessier, & Cabana, 2009; Minagawa & Wada, 1984; Pinnegar & Polunin, 1999). Different animal tissues have different turnover rates (Tieszen, Boutton, Tesdahl, & Slade, 1983) with fast turnover tissues (e.g., plasma or liver) representing short‐term diet while slow turnover tissues (e.g., muscle) represent long‐term diet (Carter, Bauchinger, & McWilliams, 2019). Consequently, muscle tissue can help identify consistent patterns in predator resource use (Carter et al., 2019; Vander Zanden, Clayton, Moody, Solomon, & Weidel, 2015).

Studies of vertebrate marine predator trophic niches and dietary specializations have focussed on elasmobranchs (Gallagher et al., 2017; Matich et al., 2011; Shiffman, Kaufman, Heithaus, & Hammerschlag, 2019; Shipley et al., 2018) and birds (Bodey et al., 2018; Patrick et al., 2014), with most studies focussing on only a few co‐occurring species. There is a lack of isotopic information on resource partitioning among co‐occurring teleost predators (Matley, Tobin, Simpfendorfer, Fisk, & Heupel, 2017), particularly in the tropics (Cameron et al., 2019). This is despite the fact that coral reefs often support a high biomass and diversity of sympatric teleost predators (Friedlander, Sandin, DeMartini, & Sala, 2010; Stevenson et al., 2007), a factor thought to increase the occurrence of dietary specialization (Araújo et al., 2011). Coral reefs, along with their predator populations, are currently experiencing unprecedented worldwide declines due to a range of anthropogenic and climate‐related stressors (Friedlander & DeMartini, 2002; Hughes et al., 2017). Given their potential stabilizing roles in food web dynamics, knowledge of sympatric reef predator trophodynamics and resource partitioning is important for predicting how reef communities will respond to change (Matich et al., 2011).

To our knowledge, no study to date has considered the isotopic niche partitioning of teleost coral reef predators across multiple, co‐occurring families. Greater understanding of spatial and intraspecific variation in predator feeding patterns is essential to predict how species will respond to fluctuations in resource availability as environments change (Matley et al., 2017; Shiffman et al., 2019). Here, we use a tri‐isotope ellipsoid approach to examine the isotopic niches of seven key teleost coral reef predator species to determine whether predator resource use varies 1) spatially and/or 2) intraspecifically, and 3) whether their isotopic niches overlap.

## 2 MATERIALS AND METHODS

### 2.1 Study site and sample collection

Fieldwork was conducted in North Malé atoll, Republic of the Maldives (N 04°26.154′, E 73° 29.902′) from January to April 2017. Sampling occurred at sites across two distinct reef areas, the inner lagoonal reefs (hereafter “inner atoll”) and atoll‐rim outer reef slopes (hereafter “outer atoll”) atoll (Figure S1).

In each area, seven reef predator species belonging to three families were sampled opportunistically: groupers (Serranidae: *Aethaloperca rogaa*, redmouth; *Anyperodon leucogrammicus*, slender; *Cephalopholis argus*, peacock; *Cephalopholis miniata*, coral hind), snappers (Lutjanidae: *Lutjanus bohar*, red; *Lutjanus gibbus*, humpback), and jack (*Caranx melampygus*, bluefin trevally). Predators (trophic level ≥ 3.5) were chosen for sampling based on their status as key fishery target species (Sattar, Wood, Islam, & Najeeb, 2014) and being dominant components of the predator assemblage biomass in both inner and outer atoll areas (first author, unpublished data). Predators were caught using rod and reel, handlines and pole spears. For each individual, the total length (cm) was recorded, and then, a sample of dorsal white muscle tissue (1–2 g wet mass) was removed. Sampling was conducted nonlethally where possible using a 4 mm biopsy punch. All tissue sampling was carried out in compliance with UK Home Office Scientific Procedures (Animals) Act Requirements and approved by the Newcastle University Animal Welfare and Ethical Review Body (Project ID No: 526). Only adults were sampled to limit possible ontogenetic dietary shifts.

Tissue samples were oven‐dried at 50°C for 24 hr, redried using a freeze drier, and then ground to a fine homogenous powder using a pestle and mortar. Subsamples of 2.5 mg of tissue were weighed into 3 × 5 mm tin capsules and sequentially analyzed for δ^15^N, δ^13^C, and δ^34^S using a PyroCube elemental analyser (Elementar, Hanau, Germany) interfaced with an Elementar VisION isotope ratio mass spectrometer at the East Kilbride (UK) node of the Natural Environment Research Council Life Sciences Mass Spectrometry Facility in August 2017. Stable isotope ratios are reported using the delta (δ) notation which for δ^13^C, δ^15^N, or δ^34^S is: $\left[\left(R_{\text{sample}}/R_{\text{standard}}\right)-1\right]$, where *R* is the ratio of the heavy to light isotope (e.g., ^13^C/^12^C), and measured values are expressed in per mil (‰).

International reference materials were placed at the start and end of each N/C/S run (~140–150 samples) to correct for accuracy and drift. Materials used were USGS40 (glutamic acid) for δ^13^C and δ^15^N (analytical precision (*SD*) δ^13^C = 0.07; δ^15^N = 0.16) and silver sulfide standards IAEA‐S1, S2, and S3 for δ^34^S (analytical precision (*SD*) = 0.17, 0.59, and 1.46, respectively). Internal reference materials were placed every 10 samples. Materials used were MSAG2 (a solution of methanesulfonamide and gelatin), M2 (a solution of methionine, gelatin, glycine), and ^15^N‐enriched alanine and SAAG2 (a solution of sulfanilamide, gelatin, and ^13^C‐enriched alanine) (Table S1). A randomly spaced study‐specific reference was also used (one mature individual [TL = 41.4 cm] of *A. leucogrammicus*, analytical precision (*SD*) δ^13^C = 0.14, δ^15^N = 0.27, and δ^34^S = 0.73, respectively, *n* = 31) (Table S1).

High lipid content in fish muscle tissue can skew carbon isotope data interpretations as lipids are depleted in ^13^C relative to proteins (Focken & Becker, 1998). Carbon stable isotope data were lipid corrected arithmetically when the C:N ratio of the muscle tissue was > 3.7 using the mass balance equation from Sweeting, Polunin, and Jennings (2006):

(1) $$\delta^{13}C_{\text{protein}}=\frac{\left(\delta^{13}C_{\text{sample}}\times C:N_{\text{sample}})+(7\times \left(C:N_{\text{sample}}-C:N_{\text{protein}}\right)\right)}{C:N_{\text{sample}}}$$

Here, C:N protein was 3.7 determined by Fry et al. (2003) from shrimp muscle protein C:N.

### 2.2 Ellipsoid metrics

The “SIBER” package in *R* (Jackson, Inger, Parnell, & Bearhop, 2011) provides methods for analyzing bivariate stable isotope data although such methods are applicable to any bivariate normally distributed data. We extend these methods to the three‐dimensional case in order to apply ellipsoids to trivariate data and calculate their overlap.

Ellipsoid volume can be estimated analytically from the sample covariance matrix by decomposition into their respective eigenvalues and eigenvectors. In the three‐dimensional case, the square root of the eigenvalues represents the three orthogonal axes, one semimajor and two semiminor (a, b, and c, respectively), that describe the standard ellipsoid, synonymous to the two‐dimensional standard ellipse (Jackson et al., 2011). The standard ellipsoid captures approximately 20% of the data (Friendly 2007), which can be subsequently rescaled to capture any desired proportion of data. The volume of the ellipsoid is then taken to be (4/3)πabc which we denote SEV. As with SEA, SEV is biased to underestimation of volume when sample sizes are small (Jackson et al., 2011). A small sample size correction for degrees of freedom following Friendly (2007) can be applied to correct for such bias giving SEV_C_, equivalent to SEAc (Jackson et al., 2011), and only here, the correction factor is (n-1)/(n-3) as the ellipsoids are in three dimensions.

To quantify uncertainty in SEV estimates, a Bayesian framework was developed by generalizing code in the SIBER package to the *n‐*dimensional case (Jackson et al., 2011). Data are assumed to be well described by the multivariate normal distribution and Bayesian posteriors of the mean and covariance structures estimated using JAGS via the R package RJAGS (Plummer, 2018). Ellipsoid volume can subsequently be estimated from each covariance draw to provide a posterior estimate of SEV, which we denote SEV_B_. Sensitivity analysis indicates that this Bayesian approach slightly underestimates population SEV at small sample sizes (approximately *n* ≤ 8, see Figure S2).

To estimate the degree of overlap between two ellipsoids, we used a numerical approach, utilizing the packages “rgl” (Adler et al., 2018) and “geometry” (Habel, Grasman, Gramacy, Mozharovskyi, & Sterratt, 2019). Ellipsoids were approximated by three‐dimensional meshes: a series of vertices that lie on the ellipsoid surface forming quadrilateral faces. The intersection of these two meshes is then approximated by a third mesh, the convex hull of which estimates the ellipsoid overlap volume. This method underestimates volumes as convex surfaces are approximated by planar faces; however, this bias is reduced as the number of vertices used to represent the ellipsoids increases, which can be iteratively increased by subdividing faces (see Figure S3). As with estimating SEV_B_, we use a Bayesian approach to estimate data covariance structures and calculate overlap for each paired posterior draw to provide a posterior estimate of overlap. Functions for estimating SEV, SEV_C_, SEV_B,_ and overlap posteriors are provided in an R script in the supplementary.

### 2.3 Data analysis: application

The ranges in carbon (CR), nitrogen (NR), and sulfur (SR) isotope values for each predator were calculated (Layman, Quattrochi, Peyer, & Allgeier, 2007). Using the MVN R Package (Korkmaz, Goksuluk, & Zararsiz, 2014), multivariate normality was checked using Mardia's test (Mardia, 1970) as it can calculate a corrected version of skewness for small sample sizes (<20). All species in each area conformed to multivariate normality (*p* > .05) with the exception of *L. gibbus* and *L. bohar* in the inner atoll. Both had normal kurtosis (*p* > .05) but were non‐normally skewed (*p* < .05). Univariate normality tests showed that δ^34^S was normally distributed for both species, δ^15^N was only normally distributed for *L. gibbus*, and both had non‐normally distributed δ^13^C. The non‐normality was driven by one *L. gibbus* with a more positive δ^13^C and two *L. bohar* that had more positive δ^13^C and lower δ^15^N, respectively. As all the other data conformed to multivariate normality and these data points represent individuals with differing resource uses (Jackson et al., 2011), data were considered well described by the multivariate normal distribution for all further analysis.

For each species in each area, Bayesian estimates for the multivariate normal distribution of the data were calculated (15,000 iterations with a burn‐in of 10,000 and a thinning factor of 25). Bayesian ellipsoids were fit to 75% of the data (EV_B_), and their median volume and interquartile range (25%–75%) were determined. The degree of ellipsoid overlap between species within each area was calculated based on EV_B_ where Bayesian posteriors were determined from 7,500 iterations with a burn‐in of 5,000 and a subdivision value of 4. Overlap was expressed as a median percentage with 95% credible intervals where 100% indicates completely overlapping ellipsoids and 0% indicates entirely distinct ellipsoids. When the overlap between two species was ≥60%, niche overlap was considered significant (Matley et al., 2017). Outer atoll *L. bohar* were excluded as only one fish was caught.

Individual body size may also influence trophic interactions; we tested for this using mixed‐effects models with the R package lme4 (Bates, Maechler, Bolker, & Walker, 2015). The δ^13^C, δ^15^N, or δ^34^S stable isotope values were the response variable, with area (inner/outer) and total length (mm) (and their interaction) as fixed effects and total length (mm) nested within species as a random effect. Model normality and homogeneity assumptions were checked by plotting model residuals. Significant effects were determined using the R package lmerTest (Kuznetsova, Brockhoff, & Christensen, 2017) which provides *p*‐values for lmer model fits via Satterthwaite's degrees of freedom method. Statistical power to detect size‐related effect was determined using the simr R package (Green & MacLeod, 2016). All analyses were carried out in R Statistical Software version 3.5.2 (R Core Team, 2017) and RStudio version 1.1.383 (RStudio Team, 2012).

## 3 RESULTS

There were substantial differences in the isotope values among the seven species sampled in both areas (Table 1). δ^13^C ranged from −18.00 (*A. rogaa*, outer) to −10.11 (*Lutjanus bohar*, inner), δ^15^N ranged from 10.11 (*L. bohar*, inner) to 14.59 (*L. gibbus*, outer), and δ^34^S ranged from 17.06 (*C. melampygus*, inner) to 21.02 (*A. rogaa*, outer).

**Table 1:** Summary information for the predators in inner and outer atoll

Family	Species	Area	*n*	Size (mm)	δ^13^C (‰)	CR	δ^15^N (‰)	NR	δ^34^S (‰)	SR
Carangidae	*Caranx melampygus*	Inner	10	248–410	−16.47 (0.22)	3.50	12.39 (0.17)	0.48	18.12 (0.15)	1.20
		Outer	6	372–461	−15.80 (0.02)	0.93	12.44 (0.20)	1.48	18.25 (0.16)	1.29
Lutjanidae	*Lutjanus bohar*	Inner	12	210–370	−15.36 (0.63)	7.06	12.36 (0.29)	2.94	18.59 (0.18)	0.70
		Outer	1	185	−14.87 (0.00)		12.97 (0.00)		17.94 (0.00)	
	*Lutjanus gibbus*	Inner	13	244–357	−16.36 (0.15)	2.96	12.58 (0.08)	0.02	19.14 (0.17)	1.51
		Outer	9	287–420	−16.26 (0.60)	7.84	12.99 (0.32)	3.54	18.96 (0.33)	2.84
Serranidae	*Aethaloperca rogaa*	Inner	11	164–278	−16.08 (0.26)	2.72	12.77 (0.07)	0.14	19.49 (0.17)	0.99
		Outer	11	148–336	−17.11 (0.17)	4.02	12.99 (0.16)	0.96	19.79 (0.18)	1.95
	*Anyperodon leucogrammicus*	Inner	10	238–346	−15.60 (0.19)	1.91	12.94 (0.11)	0.11	19.49 (0.17)	0.79
		Outer	10	262–426	−15.61 (0.04)	3.37	12.81 (0.15)	0.42	19.28 (0.01)	0.17
	*Cephalopholis argus*	Inner	11	186–342	−15.46 (0.23)	2.81	12.77 (0.08)	0.01	19.32 (0.26)	1.78
		Outer	10	190–345	−16.14 (0.19)	2.42	12.29 (0.08)	0.72	19.58 (0.14)	0.53
	*Cephalopholis miniata*	Inner	11	160–320	−16.92 (0.10)	2.87	12.73 (0.06)	0.21	19.73 (0.17)	1.47
		Outer	10	161–298	−16.88 (0.22)	4.23	12.64 (0.10)	1.26	19.55 (0.20)	0.52

Note

Mean δ^13^C, δ^15^N, and δ^34^S values are in per mil (‰) with *SE* in brackets.

Abbreviations: CR: δ^13^C range, NR: δ^15^N range, SR: δ^34^S range.

**Figure 1:**
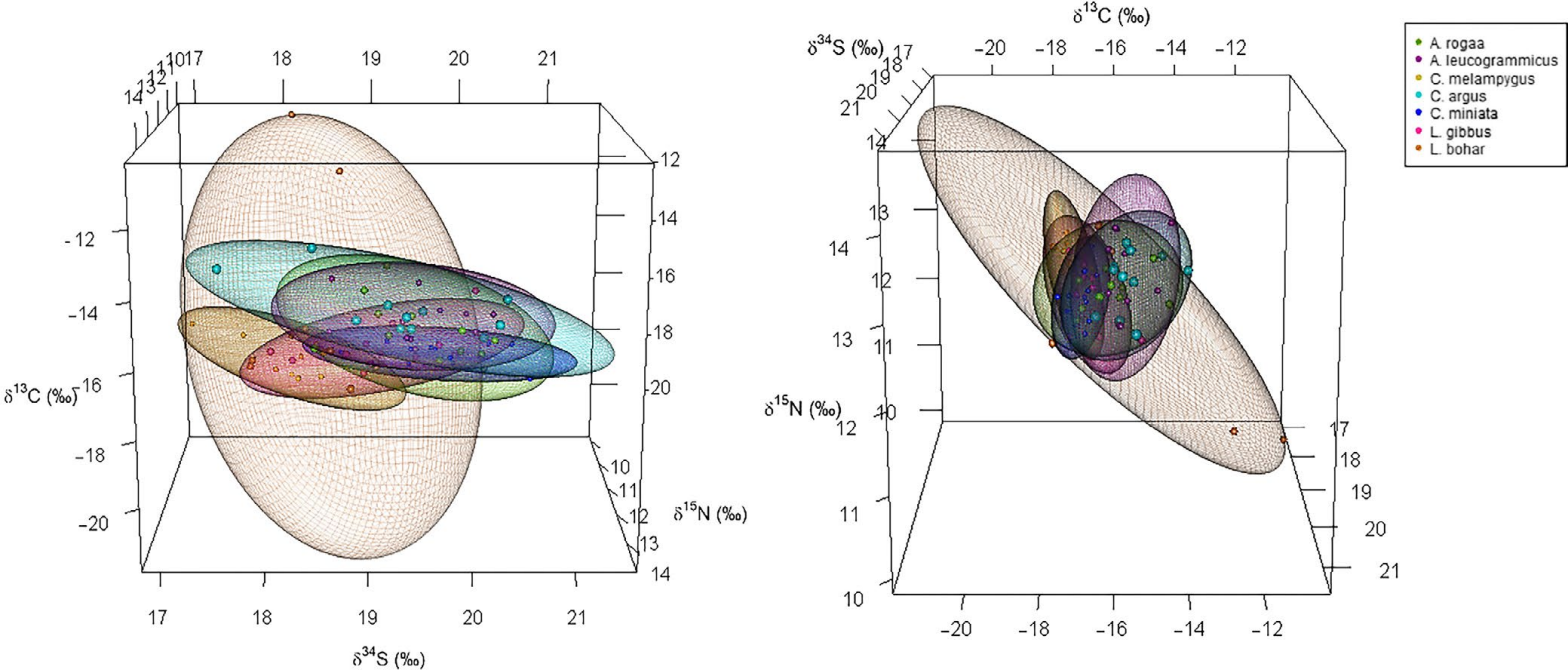
75% ellipsoids corrected for small sample size generated using δ^13^C, δ^15^N, and δ^34^S data for predators in the inner atoll

In the inner atoll, the median niche volume of *L. bohar* (25.62) was five times larger than the niches of the other predators. Excluding *L. bohar*, *C. miniata* median niche volume (3.22) was half the size of the niches of the other predators, while that of *C. argus* was double the size (8.10). *C. melampygus* and *L. gibbus* had median niche volumes that were of a similar size (4.21 and 4.76, respectively), and *A. rogaa* and *A. leucogrammicus* had niches of a similar size (6.22 and 5.53, respectively) (Table 2; Figure 1).

**Table 2:** Bayesian 75% ellipsoid volume (EV_B_) estimates for predators sampled in inner and outer atoll, given as median with interquartile range (IQR, 25th and 75th percentile)

Species	Inner		Outer	
	Median	IQR	Median	IQR
*Aethaloperca rogaa*	6.22	3.95, 6.89	6.45	4.39, 7.22
*Anyperodon leucogrammicus*	5.53	3.78, 6.30	7.96	5.27, 9.06
*Caranx melampygus*	4.21	2.85, 4.87	6.78	3.61, 7.51
*Cephalopholis argus*	8.10	5.13, 8.92	4.32	2.77, 4.69
*Cephalopholis miniata*	3.22	1.98, 3.45	7.06	4.36, 7.65
*Lutjanus bohar*	25.62	18.15, 29.14		
*Lutjanus gibbus*	4.76	3.30, 5.30	20.63	12.58, 22.67

**Figure 2:**
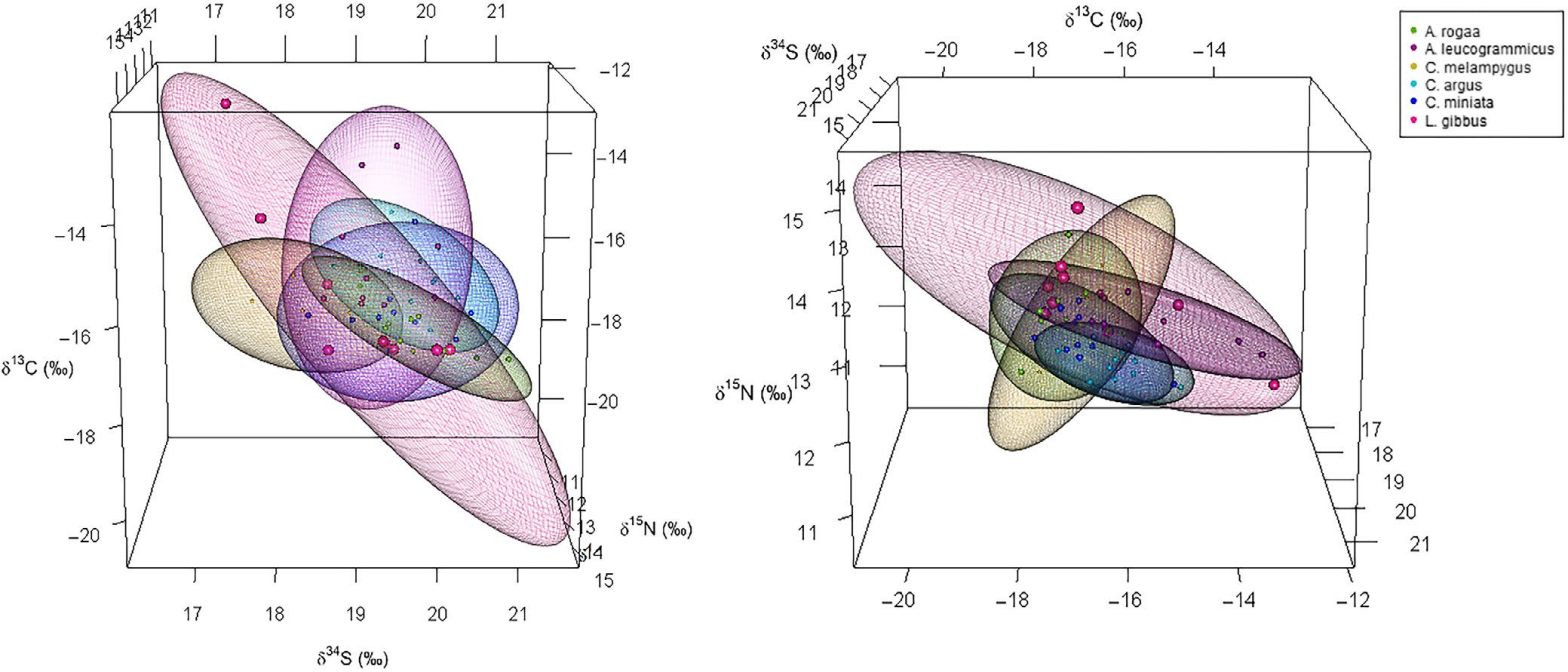
75% ellipsoids corrected for small sample size generated using δ^13^C, δ^15^N, and δ^34^S data for predators in the inner atoll

In the outer atoll, the median niche volume of *L. gibbus* (20.63) was five times larger than the niches of the other predators. The niche volumes of all the other predators were of similar size (6.45–7.96), except for *C. argus* which had the smallest median niche volume (4.32) (Table 2; Figure 2).

All predators had larger median isotopic niche volumes in the outer atoll than in the inner atoll, except for *C. argus* (inner: 8.10; outer: 4.32) (Table 2; Figure 1 and Figure 2). Median niche volume of *L. gibbus* in the outer atoll (20.63) was four times larger than the niche volume of their inner atoll conspecifics (4.76). *C. miniata* had a median niche volume twice as large in the outer atoll (inner: 3.22; outer: 7.06), while the niches of *A. leucogrammicus* and *C. melampygus* were only 1.5 times larger in the outer atoll (Table 2; Figure 1 and Figure 2).

There were no effects of body size or area on predator δ^15^N and δ^34^S values (Table S2) but statistical power was low (Median [95% CI] δ^15^N: 9% [4–16] and δ^34^S: 14% [8–22]). Statistical power to detect size effects was highest for δ^13^C (Median (95% CI) δ^13^C: 70% (60 – 77)) but there were no overall size effects on predator δ^13^C values. However, they were significantly more negative in the outer atoll (*p* < .01) and there was a significant effect of size interacting with area (*p* < .05) (Table S2).

There were few instances of significant niche overlap among the predators in the inner atoll. *A. leucogrammicus* had a niche that significantly overlapped with *C. argus* (median overlap: 63%), and *L. gibbus* had a niche that significantly overlapped with *L. bohar* (median overlap: 74%) (Table 3). There were no instances of significant niche overlap among predators in the outer atoll (Table 3).

**Table 3:** Median percentage overlap in ellipsoids (Bayesian 75% ellipsoid generated using δ^13^C, δ^15^N, and δ^34^S data) with 95% credible intervals showing the uncertainty in the overlap estimates between each pair of predator species

		*A. rogaa*	*A. leu*	*C. mel*	*C. argus*	*C. miniata*	*L. bohar*	*L. gibbus*
Inner	*A. rogaa*	—	46 (18–77)	1 (0–14)	57 (24–86)	30 (11–52)	39 (11–78)	31 (9–57)
	*A. leu*	53 (24–85)	—	0 (0–4)	**63 (33–95)**	12 (0–29)	18 (0–52)	16 (0–39)
	*C. melampygus*	2 (0–20)	0 (0–5)	—	0 (0–8)	5 (0–23)	57 (30–94)	29 (7–56)
	*C. argus*	45 (20–75)	42 (18–70)	0 ( 0–4)	—	10 (0–26)	30 (8–64)	14 (0–31)
	*C. miniata*	57 (25–94)	21 (0–56)	6 (0–30)	27 (0–64)	—	46 (13–85)	53 (24–86)
	*L. bohar*	10 (2–23)	4 (0–12)	10 (3–20)	10 (2–23)	6 (1–14)	—	14 (5–26)
	*L. gibbus*	41 (15–70)	18 (0–42)	26 (6–50)	24 (0–50)	36 (12–61)	**74 (48–100)**	—
Outer	*A. rogaa*	—	29 (7–59)	10 (0–35)	20 (2–44)	47 (22–79)	—	56 (25–89)
	*A. leu*	23 (5–43)	—	9 (0–32)	16 (1–38)	26 (4–54)	—	51 (20–82)
	*C. melampygus*	10 (0–34)	12 (0–36)	—	3 (0–19)	17 (0–47)	—	34 (7–69)
	*C. argus*	31 (5–61)	31 (5–65)	5 (0–35)	—	55 (23–90)	—	29 (2–76)
	*C. miniata*	44 (17–76)	31 (4–60)	17 (0–43)	33 (11–65)	—	—	46 (9–85)
	*L. gibbus*	18 (5–36)	20 (6–42)	11 (2–27)	7 (0–17)	16 (4–32)	—	—

Note

The table is to be read across each row: for example, in the inner atoll 46% of the *Aethaloperca rogaa* ellipsoid overlapped with the *Anyperodon leucogrammicus* ellipsoid, and 53% of the *A. leucogrammicus* ellipsoid overlapped with the *A. rogaa* ellipsoid. Significant overlap (≥60%) is in bold. Overlap was only determined for predators in the same atoll area.

## 4 DISCUSSION

This study is the first to investigate how resource use varies intraspecifically and spatially for multiple sympatric coral reef predators across an atoll seascape. To date, most studies of reef predator trophodynamics in the tropics have focussed on single species or genera, despite the multispecies nature of many coral reef fisheries (Newton, Cote, Pilling, Jennings, & Dulvy, 2007). We reveal considerable spatial variation in predator resource use inferred from variability in isotopic composition, suggesting differences within and among species.

### 4.1 Is there intraspecific variation in predator resource use?

Although considered to be generalist predators, the large variation in isotope niche volumes, as determined by the 75% Bayesian ellipsoid volume (EV_B_), suggests differences in resource utilization among species. The niches of *L. bohar* (inner atoll) and *L. gibbus* (outer atoll) were estimated to be larger than those of the other predators. For both these species, larger EV_B_ was driven by two individuals that differed considerably in isotope values from the rest (higher δ^13^C, lower δ^15^N, and δ^34^S), despite being of similar sizes to their conspecifics. As stable isotope values are time‐integrated indicators of assimilated food items, the less negative δ^13^C of these individuals indicates consistent feeding on more benthic prey. It also suggests that prey from a range of production sources are available to the predators across the atoll seascape. This hypothesis is supported by isotope values of primary consumers, which had large but similar ranges in both atoll areas (Inner δ^13^C −18.26 to −11.93; δ^15^N 6.70 to 12.39; δ^34^S 18.14 to 22.40; Outer δ^13^C −17.49 to −11.77; δ^15^N 6.24 to 11.74; δ^34^S 18.79 to 20.42) (Skinner, Newman, Mill, Newton, & Polunin, 2019b).

There is little published information on the movements of *L. bohar* and *L. gibbus* specifically; snappers generally have high site fidelity, although this can vary spatially (Farmer & Ault, 2011; Pittman et al., 2014). As such, these isotope data give insight in to their foraging behaviors in the absence of spatial tracking methods to assess resource partitioning. In the Bahamas, δ^13^C values of *Lutjanus griseus* and *Lutjanus apodus* indicated consistent intraspecific variability in space and resource use, with some individuals exploiting different areas of a creek and more marine‐based resources, while others did not (Hammerschlag‐Peyer & Layman, 2010). In our Maldives data, some individuals of *L. bohar* and *L. gibbus* appeared to be feeding on more benthic prey (less negative δ^13^C) at lower trophic levels (lower δ^15^N). Stomach content data indicate that both *L. bohar* and *L. gibbus* are capable of feeding on a range of prey, foraging predominantly on reef‐associated fish but also partly on crustaceans (Randall & Brock, 1960; Talbot, 1960; Wright, Dalzell, & Richards, 1986). The isotopic differences among individuals sampled within the same area suggest they may have alternative feeding strategies focusing on different prey. This specialization within populations may explain how coral reefs can support a high density of co‐occurring predators.

### 4.2 Is there spatial variation in predator resource use?

Community‐wide isotope metrics (Layman, Arrington, Montan, & Post, 2007) suggested that all four grouper species (*A. rogaa*, *A. leucogrammicus*, *C. argus*, and *C. miniata*) varied in their resource use spatially. All four had larger NR values in the outer atoll, and with the exception of *C. argus*, they all had larger CR values in the outer atoll. Although δ^15^N values of a corallivore, *Chaetodon meyeri*, and a nocturnal planktivore, *Myripristis violacea*, were significantly higher in the outer atoll, the differences in mean values were small (~1‰) and isotopic values of all other prey species were similar between areas (Skinner et al., 2019b). Furthermore, δ^13^C and δ^15^N values of coral host and particulate organic matter (POM) are consistent around the Maldives and do not vary between inner and outer atoll (Radice et al., 2019). This suggests that the differences in predator CR and NR ranges are a direct result of feeding on different combinations of prey, rather than differences in baseline isotope values.

Stomach content data show that *A. rogaa*, *C. argus*, and *C. miniata* feed primarily on reef‐associated fish from a range of families that are sustained by multiple production sources (Dierking, Williams, & Walsh, 2011; Harmelin‐Vivien & Bouchon, 1976; Shpigel & Fishelson, 1991). While no stomach content data were available for *A. leucogrammicus*, it likely has a similar diet to the other groupers. The larger CR and NR of these species could indicate that their prey rely on a wide range of production sources. Where benthic and pelagic food webs overlap such as here, predators might have access to prey from two food webs (i.e., planktivores and herbivores) while remaining in the same habitat (Matich et al., 2011). Furthermore, *C. argus* in particular displays extensive foraging plasticity allowing it to take advantage of small scale fluctuations in prey availability (Karkarey, Alcoverro, Kumar, & Arthur, 2017), a behavior possibly reflected in the larger CR and NR ranges.

Interestingly, and in contrast to the patterns identified with the CR and NR ranges, *A. leucogrammicus* and *C. miniata* had larger SR ranges (δ^34^S: 18.60–20.29 and 18.70–20.65, respectively) in the inner atoll, despite having smaller CR and NR ranges and isotopic niches there. The δ^34^S isotope values revealed that these two species may be feeding on prey reliant on a range of production sources, including more benthic‐sustained detritivores (mean ± *SD* δ^34^S: 18.14 ± 0.22) and herbivores (mean ± *SD* δ^34^S: 19.66 ± 0.22) (Skinner et al., 2019b). Assessing the resource use of these two inner atoll predators solely based on δ^13^C and δ^15^N values may have missed this intricacy, as the δ^13^C and δ^15^N values were indicative of feeding on more pelagic prey from higher trophic levels (evidenced by lower δ^13^C and higher δ^15^N). In food web studies, δ^34^S is often overlooked, despite its ability to help distinguish between different marine producers (Connolly et al., 2004) and reveal resource usage intricacies and pathways (Croisetière et al., 2009; Gajdzik, Parmentier, Sturaro, & Frédérich, 2016) that may be masked using only δ^13^C or δ^15^N. The primary reason for this is that measuring δ^34^S is typically more challenging, and thus more costly, than measuring δ^13^C or δ^15^N. However, recent technological advances and new instruments mean that δ^13^C, δ^15^N, and δ^34^S can be measured from the same sample aliquot with a high level of precision (Fourel, Lécuyer, & Balter, 2015). Given these advances and the relative ease of measuring δ^34^S, we strongly suggest that more studies incorporate δ^34^S to employ the tri‐isotope ellipsoid approach that we present here.

With the exception of *C. argus*, all predators had larger isotopic niches in the outer atoll. Given the similarity in prey and primary producer isotope values between atoll areas (Radice et al., 2019; Skinner et al., 2019b), it seems likely that this spatial variation in resource use is linked to variations in resource availability (Araújo et al., 2011). The oceanic rim reefs of the outer atoll had higher live branching coral and habitat structural complexity following the 2016 bleaching event compared with inner atoll reefs (first author, unpublished data). Coral cover is strongly linked to fish species richness (Komyakova, Munday, & Jones, 2013), and reefs with higher complexity and coral cover support greater densities of smaller‐bodied (<20 cm) fish (Alvarez‐Filip, Gill, & Dulvy, 2011). Although prey fish biomass was similar between atoll areas, densities of planktivores were greater along the outer edge reefs (first author, unpublished data). This may lead to increased specialization and population niche size, a hypothesis supported by the larger isotopic niche volumes of the predator populations in the outer atoll.

Inner atoll *L. gibbus* had an isotopic niche volume (EV_B_) a tenth the size of the outer atoll population. Spatial differences in *L. gibbus* feeding have previously been recorded; it has a crab‐dominated diet in Japan (Nanami & Shimose, 2013) but a forage fish (clupeid)‐dominated diet in Yemen (Ali, Belluscio, Ventura, & Ardizzone, 2016). Differential preferences for crabs, which are benthic, and clupeids, which are pelagic, may explain the differing range in δ^13^C and δ^34^S values between atoll areas found here. Furthermore, the smaller EV_B_ of the inner atoll population may mean individuals are consistently feeding on a similar but select group of prey. As isotope values of key prey species were similar in both atoll areas (Skinner et al., 2019b), this further supports the hypothesis that there is spatial variation in resource availability across the atoll.

### 4.3 Do the isotopic niches of sympatric predators overlap?

The degree of niche overlap was low; there were only two occurrences of significant niche overlap in the inner atoll and none in the outer atoll. This might suggest that the level of competition among these species is low in both areas with predators feeding on a variety of different resources. Overlapping niches do not conclusively equate to increased competition for resources (Gallagher et al., 2017; Layman et al., 2012). All predators had a larger degree of niche overlap with *Lutjanus bohar* (inner) and *L. gibbus* (outer) due to the exceptionally large niches of these two species, but the level of direct competition may be lower. Predators could be feeding on prey over different spatiotemporal scales, which would reduce their direct competition. Alternatively, due to protein turnover and prey isotope signature integration into muscle tissue over time, predators may be feeding on ecologically different diets but still express similar isotope values, confounding interpretation of the level of competition existing in the community.

It is worth noting that not all predators caught in the same location necessarily derive their nutrition from that locality though. The bluefin trevally, *C. melampygus*, had a distinct isotopic niche which overlapped minimally with the niches of the other predators in both atoll areas. *C. melampygus* is a transient, midwater predator with an extensive territory (Holland, Lowe, & Wetherbee, 1996; Sancho, 2000) and is the most mobile of all the predators sampled. It regularly makes crepuscular migrations of 1–2 km between different habitats (Meyer & Honebrink, 2005). Furthermore, it was the only predator to occupy a similar isotopic niche in both areas, suggesting it may use resources from across the atoll. Stomach content data indicate it feeds predominantly on nekton spanning multiple trophic levels, with little reliance on crustaceans or cephalopods (Meyer, Holland, Wetherbee, & Lowe, 2001). Consequently, this separation is likely attributable to differing habitat usage and prey encounters compared to the other more reef‐associated and site‐attached species (Sluka & Reichenbach, 1995).

Ontogenetic shifts in feeding strategies are well documented (Kimirei et al., 2013; Werner & Gilliam, 1984), but adults may also vary in their resource use as a function of their size. Here, body size did not appear to drive niche variability; there was no relationship between body size and δ^13^C, δ^15^N, or δ^34^S. Although there was a significant relationship between δ^13^C and the interaction between area and body size, the effect was weak. However, statistical power was low and the ability to detect relationships may have been limited due to small sample sizes and limited size ranges; size‐based shifts in feeding might have been observed with greater replication. While more depth is needed in these data, it seems size‐based effects on adult predator resource use are absent or weak here (Gallagher et al., 2017; Layman, Winemiller, Arrington, & Jepsen, 2005; Matley et al., 2017; Shipley et al., 2018). Within the diverse food webs of coral reefs where prey sizes vary, strong relationships with body size may be masked as predators target large primary consumers (Layman et al., 2005).

Predators are often thought to be dietary generalists but we show inter‐ and intraspecific differences in resource use with minimal significant niche overlap, highlighting how trophic resource use varies among sympatric reef predators at a scale of tens of kilometers. We did not specifically test for individual specialization but several individuals of *Lutjanus* appeared to be feeding in completely different ways to their conspecifics. Individual specialization is not ubiquitous in marine predator populations (Matich et al., 2011), but small sample sizes of these predators mean statistical power to detect potential differences was limited, thus underestimating intraspecific trophic variation. Feeding specializations are linked to ecological opportunity and are thought to be more common where resource diversity and density of competing individuals are greater (Araújo et al., 2011). This makes coral reefs a prime location for predators to demonstrate vastly different individual feeding behaviors. Predators may provide stability to communities by linking separate food chains (McCann et al., 2005; Rooney et al., 2006), but individual dietary specializations could alter this ecological linkage role (Matich et al., 2011) with potential consequences for ecosystem resilience. Detailed information on individual predator resource use is required to identify their ecological role and help understand how they will respond to environmental change.

## CONFLICT OF INTEREST

None declared.

## AUTHOR CONTRIBUTIONS

CS, ACM, SPN, and NVCP conceived the ideas, all authors designed the methodology, CS collected the data, CS and JN processed the samples, CS and MRDC analyzed the data, CS led the writing of the manuscript, and all authors contributed to revisions.

## Data Availability

Data used in this study are available from the Dryad Digital Repository: https://doi.org/10.5061/dryad.7jj53hb (Skinner, Newman, Mill, Newton, & Polunin, 2019a).
