# Supplementary figures and images for: Novel tri‐isotope ellipsoid approach reveals dietary variation in sympatric predators
Source: Ecol Evol. 2019 Nov 4;9(23):13267–77. doi: 10.1002/ece3.5779 (PMC6936247; doi:10.1002/ece3.5779)

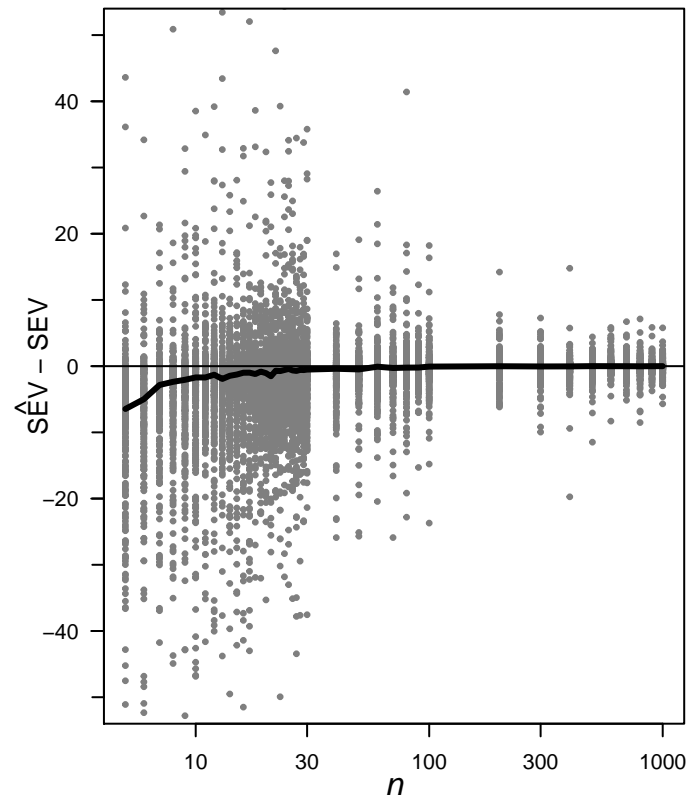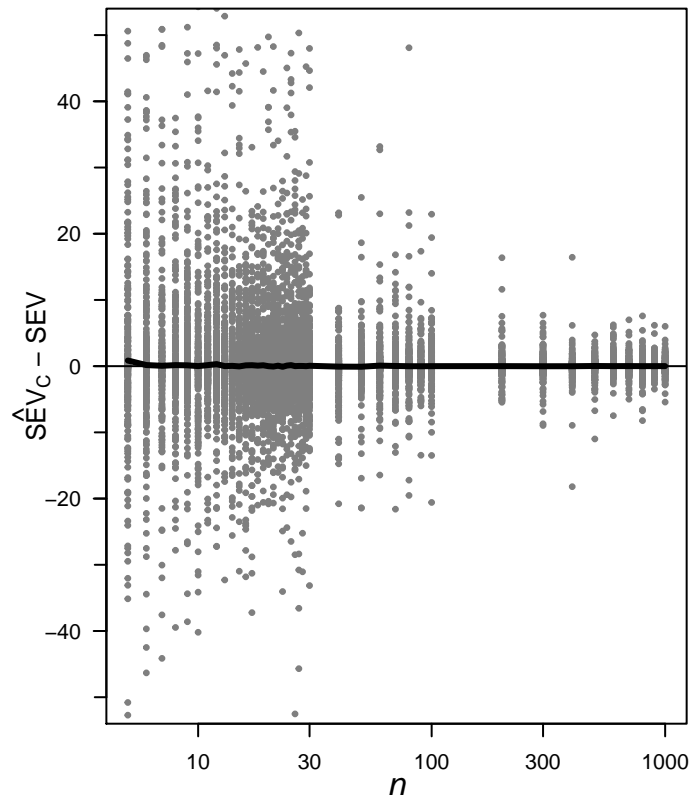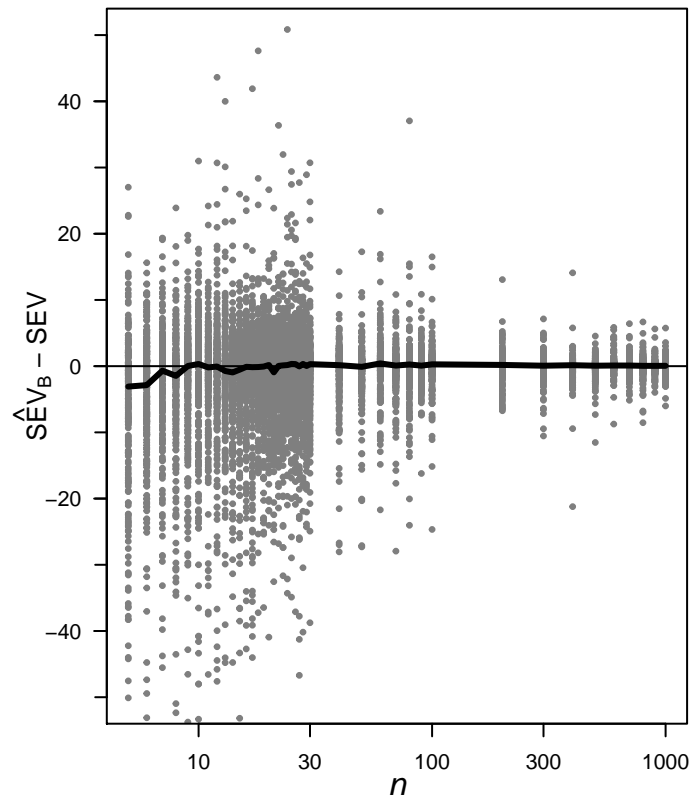

Supplement: Supplementary file 2 [file ECE3-9-13267-s002.pdf]

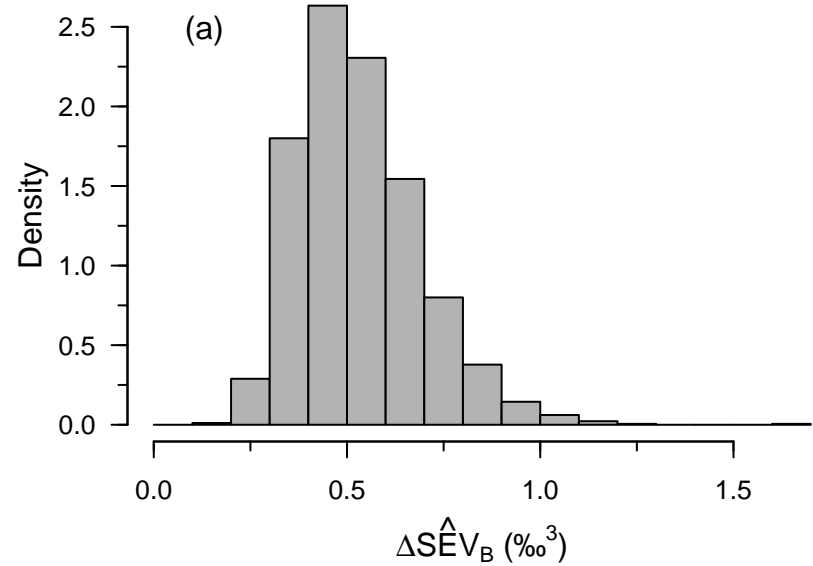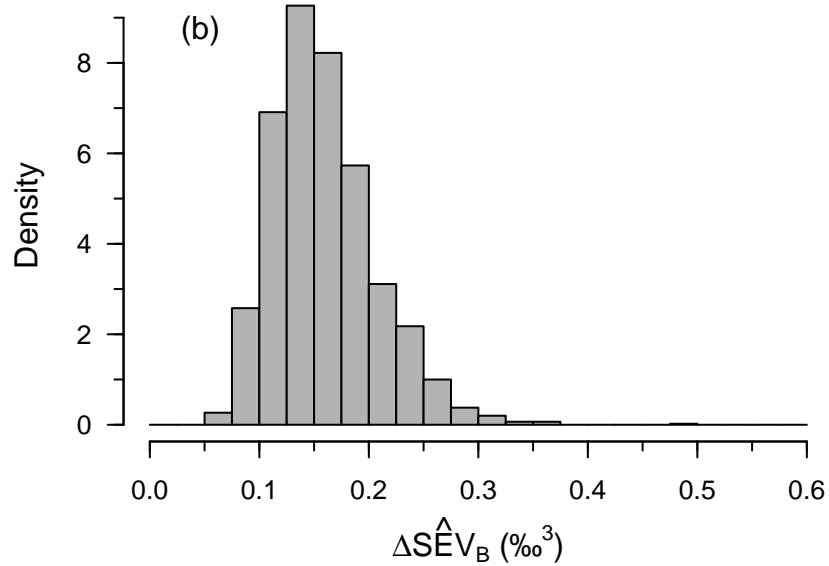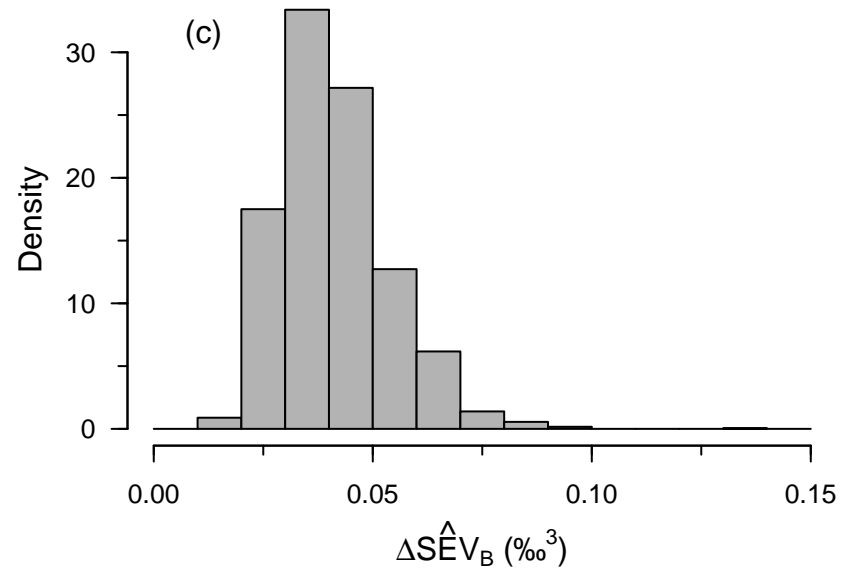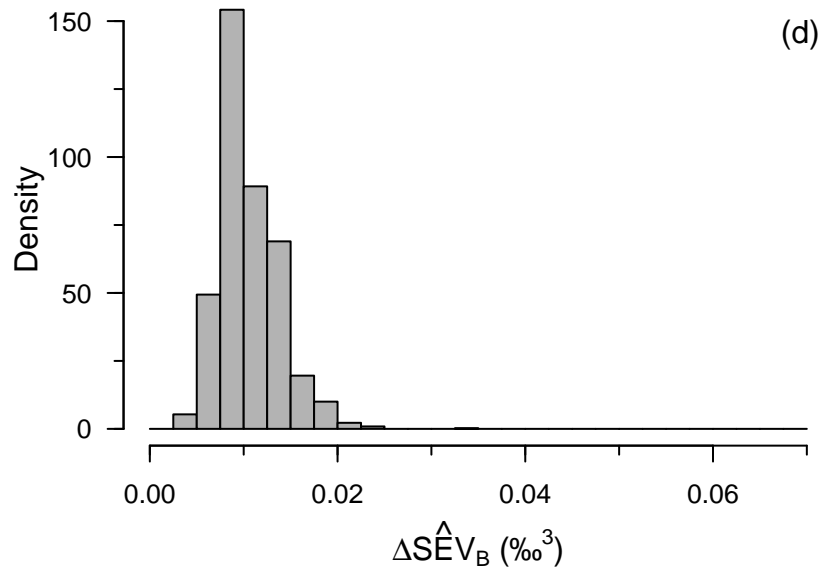

Supplement: Supplementary file 3 [file ECE3-9-13267-s003.pdf]
