# Supplementary material for: Novel tri‐isotope ellipsoid approach reveals dietary variation in sympatric predators
Source: Ecol Evol. 2019 Nov 4;9(23):13267–77. doi: 10.1002/ece3.5779 (PMC6936247; doi:10.1002/ece3.5779)
Supplement: Supplementary file 4 [file ECE3-9-13267-s004.docx]

**APPENDIX**

**Table S1**. Accepted and measured values ± SD of the international, internal and study-specific reference materials.

|  |  |  | Accepted values | | |  | Measured values | | |
| --- | --- | --- | --- | --- | --- | --- | --- | --- | --- |
| Reference | Material |  | *δ*^15^N | *δ*^13^C | *δ*^34^S | n | *δ*^15^N | *δ*^13^C | *δ*^34^S |
| International | IAEA-S1 | Mean |  |  | -0.3 | 11 |  |  | 0.01 |
|  |  | SD |  |  | * |  |  |  | 0.17 |
| International | IAEA-S2 | Mean |  |  | 22.62 | 12 |  |  | 22.16 |
|  |  | SD |  |  | 0.20 |  |  |  | 0.59 |
| International | IAEA-S3 | Mean |  |  | -32.49 | 12 |  |  | -31.61 |
|  |  | SD |  |  | 0.20 |  |  |  | 1.46 |
| International | USGS40 | Mean | -4.5 | -26.39 |  | 12 | -4.52 | -26.37 |  |
|  |  | SD | 0.1 | 0.04 |  |  | 0.16 | 0.07 |  |
| Internal | M2 | Mean | 32.70 | -34.28 | 14.43 | 78 | 32.33 | -33.82 | 13.92 |
|  |  | SD | 0.27 | 0.11 | 0.46 |  | 3.19 | 3.24 | 1.67 |
| Internal | MSAG2 | Mean | 2.24 | -21.23 | 6.18 | 116 | 2.21 | -21.36 | 6.20 |
|  |  | SD | 0.09 | 0.12 | 0.43 |  | 0.23 | 0.09 | 0.53 |
| Internal | SAAG2 | Mean | 4.55 | -5.78 | 0.04 | 65 | 4.64 | -5.67 | 0.38 |
|  |  | SD | 0.06 | 0.12 | 0.36 |  | 0.10 | 0.10 | 0.52 |
| Study-specific | *Anyperodon leucogrammicus* | Mean |  |  |  | 31 | 12.53 | -13.40 | 19.80 |
|  |  | SD |  |  |  |  | 0.27 | 0.14 | 0.73 |
| * = exact value defining scale | |  |  |  |  |  |  |  |  |

**Table S2**. Linear mixed effects models of differences in predator *δ*^13^C, *δ*^15^N and *δ*^34^S isotope values with body size and between atoll areas. The number presented is the model coefficient with the standard error in brackets. Significance level is denoted using asterisks where *** = p < 0.001, ** = p < 0.01 and * = p < 0.05.

| Formula: Isotope ~ Size + Area + Area * Size + (1 + Size \| Species) | | | |
| --- | --- | --- | --- |
|  | ***δ*^13^C** | ***δ*^15^N** | ***δ*^34^S** |
| (Intercept) | -14.63 (0.67)^***^ | 12.19 (0.38)^***^ | 18.51 (0.42)^***^ |
| Size | -0.01 (0.00) | 0.00 (0.00) | 0.00 (0.00) |
| Area | -2.25 (0.85)^**^ | -0.30 (0.43) | 0.04 (0.53) |
| Size:Area | 0.01 (0.00)^*^ | 0.00 (0.00) | -0.00 (0.00) |
| AIC | 435.74 | 254.56 | 315.78 |
| Num. obs. | 135 | 135 | 135 |

**Figure. S1**. Fish tissue sampling sites in North Malé atoll, Republic of the Maldives. Fish sampling sites were located in either the inner lagoonal reefs (inner) or along the outer edge reefs (outer).

**Figure. S2.** Bias estimation plots for population standard ellipsoid volume (SEV) as a function of sample size (n, on log scale) based on $\hat{SEV}$ (left hand plot), after small sample size correction $\hat{SEV}_{C}$(middle plot), and the Bayesian estimation $\hat{SEV}_{B}$ taken as the median posterior value (right hand plot) following methods described by Jackson *et al.* (2011). Note that the y-axis is restricted for clarity leaving some extreme values outside the depicted boundaries. Grey point are the results of 10000 total simulations, with heavy black line the median value for a given n. Thin black line shows perfect estimate of y = 0. Populations were defined by drawing from a Wishart distribution with degrees of freedom ρ = 3 and the scale matrix *V* = $\left[ \begin{matrix} 2 & 0 & 0 \\ 0 & 2 & 0 \\ 0 & 0 & 2 \end{matrix} \right]$ using the ‘MASS’ package in *R* (Venables & Ripley 2002; R Core Team 2017). Bayesian posteriors were determined from 15000 iterations with a burn in of 10000 and a thinning factor of 25.

**Fig. S3.** Density histograms of difference in overlap volume calculated from 75% $\hat{SEV}_{B}$ for *A. rogaa* and *A. leucogrammicus* data (15000 iterations with a burn in of 10000 and a thinning factor of 25) with increasing number of subdivisions used for mesh approximation of ellipsoids: 1 to 2 (a); 2 to 3 (b); 3 to 4 (c); and 4 to 5 (d). Differences rapidly converge to zero beyond 4 subdivisions. Note that both the x and y axes differ for each plot. Mesh construction and overlap approximation done using the packages ‘rgl’ (Adler, Murdoch & others 2018) and ‘geometry’ (Habel *et al.* 2019) respectively in *R* (R Core Team 2017), see code provided.

**REFERENCES**

Adler, D., Murdoch, D. & others (2018) rgl: 3D Visualization Using OpenGL. R package version 0.99.16. <https://CRAN.R-project.org/package=rgl>.

Habel, K., Grasman, R., Gramacy, R.B., Mozharovskyi, P. & Sterratt, D.C. (2019) geometry: Mesh Generation and Surface Tessellation. R package version 0.4.2. <https://CRAN.R-project.org/package=geometry>.

Jackson, A.L., Inger, R., Parnell, A.C. & Bearhop, S. (2011) Comparing isotopic niche widths among and within communities: SIBER - Stable Isotope Bayesian Ellipses in R. *Journal of Animal Ecology,* **80,** 595-602. 10.1111/j.1365-2656.2011.01806.x.

R Core Team (2017) R: A language and environment for statistical computing. R Foundation for Statistial Computing, Vienna, Austria.

Venables, W.N. & Ripley, B.D. (2002) *Modern Applied Statistics with S.,* Fourth Edition. edn. Springer, New York.
