# Supplementary material for: Novel tri‐isotope ellipsoid approach reveals dietary variation in sympatric predators
Source: Ecol Evol. 2019 Nov 4;9(23):13267–77. doi: 10.1002/ece3.5779 (PMC6936247; doi:10.1002/ece3.5779)
Supplement: Supplementary file 6 [file ECE3-9-13267-s006.xhtml]

xml version="1.0" encoding="UTF-8"?
- no title specified 
